# Extraosseous Multiple Myeloma: Case Report of Presentation in the Lower Extremity Soft Tissues with Literature Review

**DOI:** 10.1155/2017/9159035

**Published:** 2017-12-17

**Authors:** Jeff Ames, Ahmad Al-Samaraee, Takashi Takahashi

**Affiliations:** ^1^Department of Radiology, University of Minnesota, Minneapolis, MN, USA; ^2^Musculoskeletal Division, Department of Radiology, University of Minnesota, Minneapolis, MN, USA

## Abstract

A rare presentation of extramedullary multiple myeloma in the soft tissues of the bilateral thighs prompted a literature review of published cases of extramedullary multiple myeloma (EM-MM) and solitary plasmacytomas to determine the relative anatomic distribution of these lesions. All available published cases in English were included in the analysis, dating back to 1966 and including 2,538 extramedullary myeloma or solitary plasmacytoma lesions. Analysis of the anatomic location of EM-MM lesions demonstrates the majority being in the upper airway (33.8%), soft tissues including retroperitoneum and abdomen (14.1%), gastrointestinal tract (10.3%), central nervous system, head and neck (16.0%), and GU (2.4%). We were able to find only 44 documented cases of extremity soft tissue lesions, comprising 1.7% of all lesions.

## 1. Introduction

Solitary plasmacytomas and multiple myeloma can be thought of as a spectrum of disease, which ranges from localized clonal plasma cell infiltration to multiple extramedullary lesions, and osseous forms typically progress to multiple myeloma. Multiple myeloma is the most common primary osseous malignancy in adults, typically between the ages of 50 and 70, and it is more likely to affect men [1]. Multiple myeloma is defined by >10% of clonal plasma cells in bone marrow or biopsy-proven extramedullary plasmacytoma and by the evidence of end-organ damage including bone lesions and renal insufficiency [2].

The importance of diagnosing patients with atypical presentations cannot be understated as approximately 10–20% of multiple myeloma patients do not present with lytic lesions on radiography [3], and as a result MRI and PET/CT have become increasingly important in the diagnosis of multiple myeloma. It is also important to note that incidence of extramedullary myeloma is found to be 6–8% of newly diagnosed multiple myeloma patients [2]. The prevalence increases to 10–30% in relapsed/refractory patients. The disease remains incurable; however improving diagnosis and therapies have led to increasing length of survival, which has in turn increased the prevalence of atypical disease progression or features of relapse, such as extramedullary lesions [4].

## 2. Case Presentation

The patient was a previously healthy 51-year-old man who presented to the Emergency Department with back pain which he initially believed to be a pulled muscle but did not improve after three weeks. He was given pain medications, and he later followed up with his primary care physician, who ordered an MRI of his thoracic spine. The thoracic spine MRI showed diffuse marrow abnormality involving the entire thoracic spine, including anterior and posterior elements as well as visualized portions of the ribs. There was also epidural extension of the tumor at T5 level resulting in relatively severe central stenosis and displacement of the thoracic cord. Baseline imaging consisted of a bone survey which demonstrated numerous myelomatous lesions throughout the axial and appendicular skeleton.

Laboratory studies at that time demonstrated hypercalcemia and elevated creatinine, concerning for myeloma. He was started on a course of steroids with Velcade and Revlimid, and involved field radiation therapy to the thoracic spine was started with a total dose of 3000 cGy given in 10 fractions. Serum protein electrophoresis demonstrated an M-spike of 0.8 g/dL within IgG lambda. Bence Jones proteinuria was present in the range of 2 g. Biopsy of his bone marrow was consistent with aggressive myeloma with elevated plasma cells (18%), and a thorough cytogenetic evaluation demonstrated multiple abnormalities, including monosomy 13, t(4;14), deletion of 17p and 13q, and gain of 1q21. The patient was stage III (both ISS and R-ISS). He was seen shortly after diagnosis for evaluation of stem cell transplantation, and, given such a high risk of his underlying myeloma, the patient was recommended to consider allogeneic stem cell transplantation from an alternative donor source. He was also evaluated for a second opinion at an outside institution, which also recommended allogeneic stem cell transplantation in the first complete remission.

The patient completed induction therapy with VRD and achieved complete response. He underwent fludarabine-cyclophosphamide-antithymocyte antiglobulin preparation prior to a single cord blood allogeneic transplant. A repeat bone marrow biopsy following transplant did not show any evidence of multiple myeloma, and there was 91% donor chimerism. Immunosuppression leads to numerous episodes of pneumonia, and his course was complicated by renal insufficiency and acute grade III graft versus host disease, which was successfully treated with a steroid taper.

**Figure 1:**
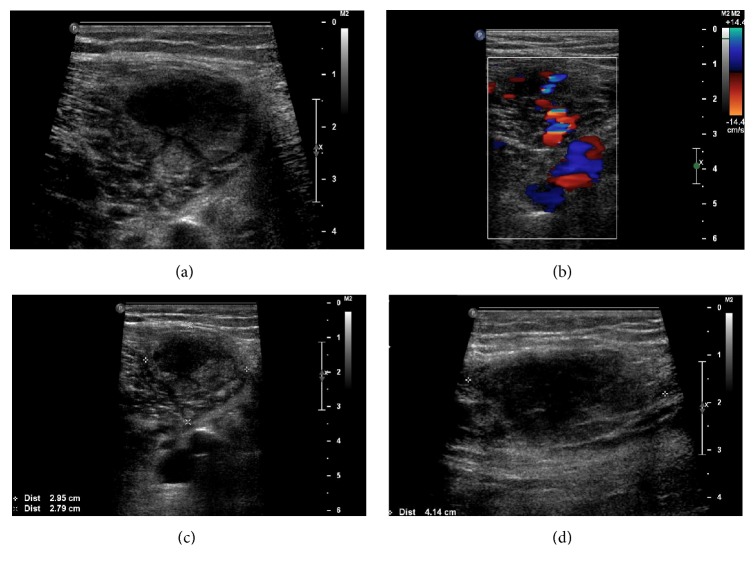
Gray scale images in transverse (a, c), longitudinal (d), and color Doppler (b) of the distal right thigh of a 51-year-old male with bilateral lower extremities pain and swelling. Images demonstrate area of heterogeneous echogenicity with internal flow in the distal right thigh. Initial interpretation was that the findings might represent muscular hematoma with possible active bleeding from overanticoagulation.

**Figure 2:**
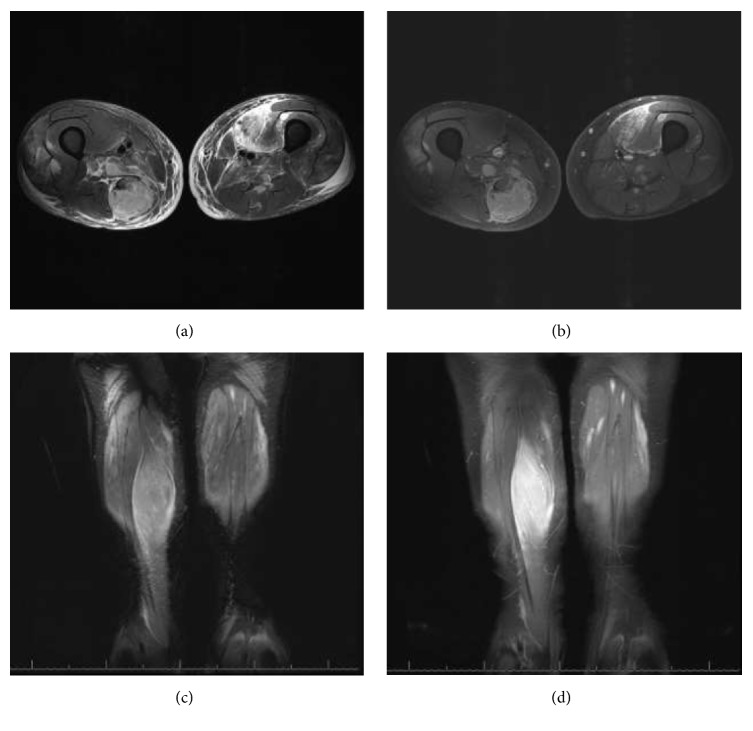
Lower extremity MRI for the same 51-year-old patient obtained a few days after the initial US. (a) is an axial T2 weighted fat suppressed image and (c) is a coronal T2 weighted fat suppressed image of the thighs. The images show multiple T2 hyperintense lesions. The largest lesion on the distal right thigh corresponds to the heterogeneous lesion seen on a week-earlier US. (b, d) are axial and coronal T1 fat suppressed postcontrast images, demonstrating associated enhancement of those lesions. Given the patient history of multiple myeloma, those lesions were suspected to represent extra-axial multiple myeloma, which was confirmed with surgical biopsy few days later.

**Figure 3:**
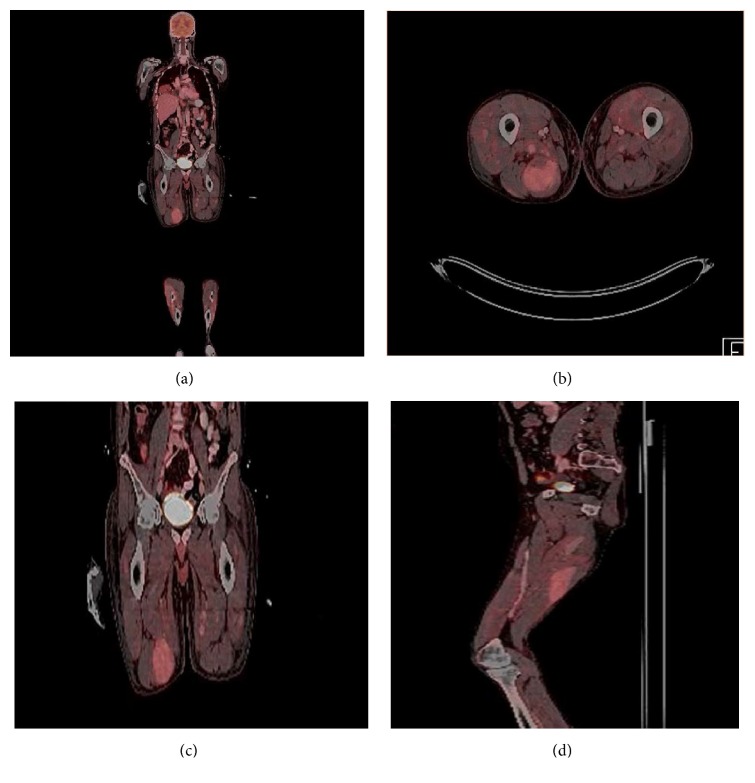
PET CT of the same 51-year-old male after approximately one week from the earlier shown US. ((a) and (c)) are coronal images of the whole body (a) and thighs (c) showing multiple foci of increased FDG uptake. (b) is an axial image of the distal thighs showing multiple foci of increased FDG uptake; the largest lesion on the posterior right thigh has SUV max of 4.3. (d) is a sagittal view of the same lesion showing its craniocaudal extension. Lesions were initially presumed and later biopsy proven to represent extra-axial multiple myeloma.

The patient was admitted approximately 5 months after transplantation for bilateral lower extremity swelling, multiple deep venous thrombi, and concern for bilateral thigh swelling. The thigh swelling was initially evaluated with ultrasound, which was read as possibly representative of hematoma in the setting of anticoagulation (Figure 1(a)). An MRI was obtained of the patient's thighs, which demonstrated extensive soft tissue enhancement bilaterally concerning for extraosseous multiple myeloma rather than hematoma (Figure 2). These soft tissue masses were biopsy-proven extramedullary plasmacytomas. Overall, the bone marrow biopsy findings were most consistent with recurrent/residual low-level multiple myeloma. Finally, a PET/CT revealed extensive osseous lesions, pulmonary involvement with pleural effusion, and numerous intramuscular lesions (Figure 3). His relapse was initially managed with VD-PACE. The patient's renal disease progressed rapidly in the following months, which ultimately led to his death despite salvage therapy (Thal/Dex/Velcade/Doxil).

## 3. Discussion

To further understand the relative proportion of anatomic locations of solitary plasmacytomas and multiple myeloma lesions outside of osseous structures, a literature review was conducted to capture all published extraosseous lesions. Many clinicians use the term “extramedullary,” which complicated the search as this could potentially be used for osseous lesions outside of bone marrow. All articles were assessed to ensure lesions were nonosseous. Lesions contiguous with or arising from osseous structures were not included [2, 5]. Plasma cell leukemias were not included [2]. Extramedullary disease was assumed not to arise from bone when not explicitly stated. Most articles did not distinguish between extramedullary disease found at diagnosis and relapse.

**Figure 4:**
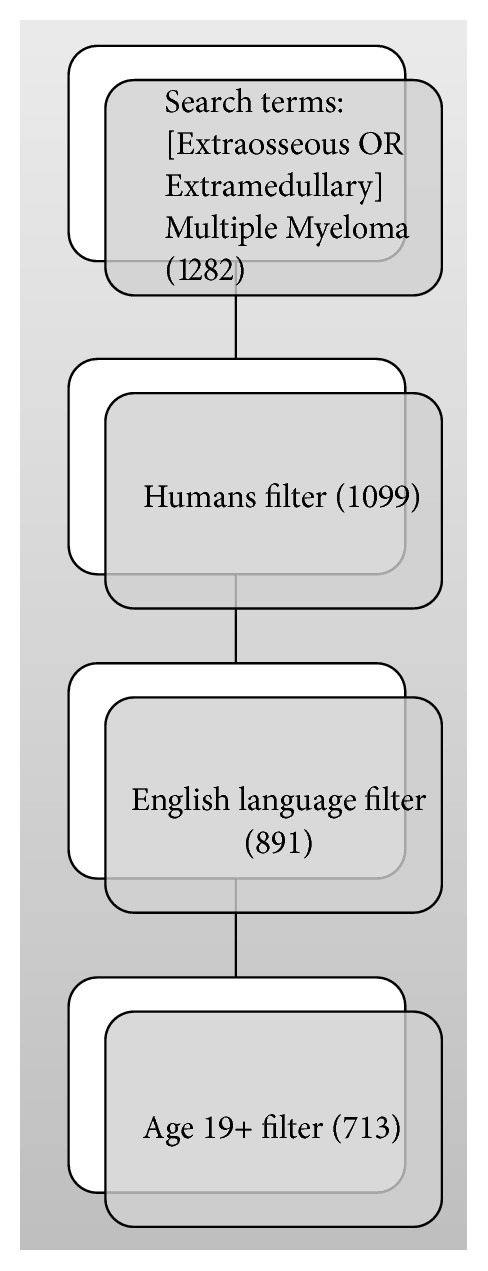
Summary of literature review (number of articles).

**Figure 5:**
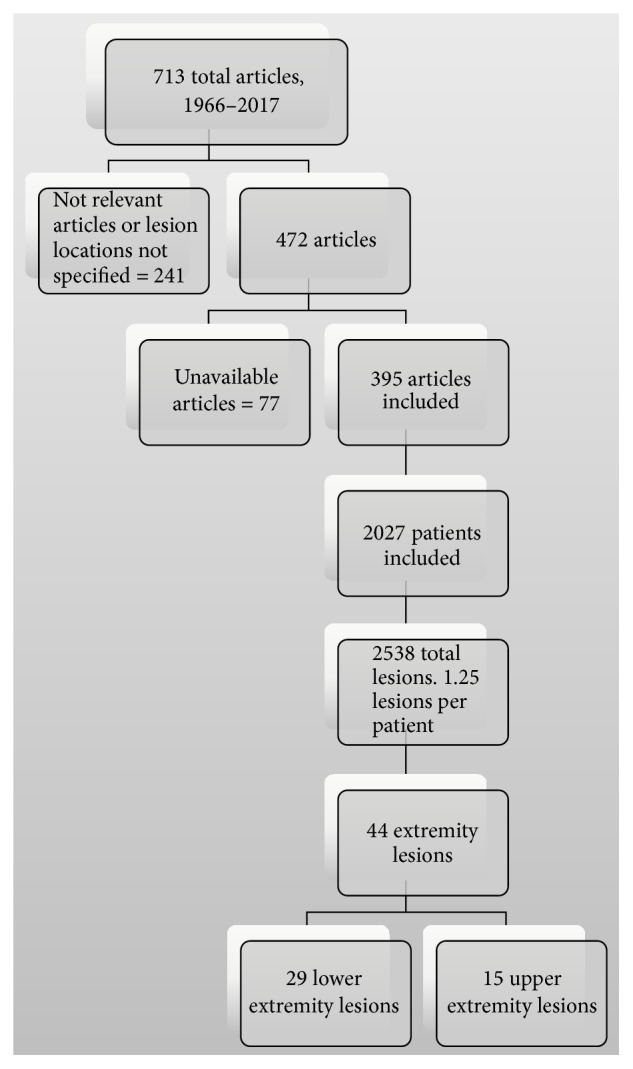
Summary of published cases.

Multifocal extramedullary disease is possible, but unless explicitly stated only the site of interest in the publication was included. PubMed was searched for “(Extraosseous OR Extramedullary) Multiple Myeloma,” and the following filters were applied: Humans, English, and All adult (19+ years old). Since 1966, there have been 713 articles published, 318 of which were excluded in the literature search as the articles were not relevant or did not specify location of extramedullary disease or access was prevented by a pay-wall (Figure 4). The included 395 articles described 2,027 patients with extramedullary disease and described 2,538 lesions (Figure 5). The majority of extramedullary lesions were in the upper airway (34%), soft tissues (14%), gastrointestinal tract (10%), or central nervous system/head and neck (16%) (Tables 1 and 2). We were able to find only 44 documented cases of extremity soft tissue lesions, comprising 2% of all lesions.

Our analysis includes all published cases of extramedullary disease, and as a result will suffer from publication bias. The anatomic distribution of extramedullary disease in the current literature review is discordant in some ways with previous retrospective/prospective analyses, for example, Usmani et al. [6]. Usmani et al. found extramedullary lesions most commonly in the abdomen/pelvis, skin/soft tissues, and paraspinal regions. We found lesions most commonly in the upper airway and oral cavity, with the central nervous system/head and neck the second most common location. The differences in anatomic distribution may be due to a larger sample size or publication bias or may represent a real change in the distribution over the past 50 years in the setting of changing therapies. Another important confounding factor is how lesions are grouped together. One feature of our analysis that was consistent with previous studies was the finding that extramedullary disease in the extremities was extremely uncommon.

Diagnosis of extramedullary disease is uncommon yet clinically important because it is associated with shorter progression-free and overall survival. The presence of extramedullary disease is also correlated with genomically high risk disease and increased rates of relapse [6]. It is also important to note that extramedullary disease can demonstrate a different genomic profile as compared to plasma cells in the bone marrow of the same patient [7]. Traditional radiography classically demonstrates osseous myeloma as lytic lesions without sclerotic margins; however these lesions are not radiographically apparent in a minority of cases which can lead to false negatives. As patients undergo repeated cycles of therapy, tumor serum markers and cross-sectional imaging are both used to determine therapy response.

Conventional radiography or bone survey has traditionally been the most common imaging modality in multiple myeloma; however there are numerous and increasing reasons why radiography alone is no longer adequate. Extramedullary nonosseous disease will not be visualized radiographically and as myeloma patients are living longer, extramedullary disease becomes more common and subsequently cross-sectional radiological evaluation should be considered, including MRI or PET/CT. Additionally, a systematic review performed by Regelink et al. in 2013 demonstrated greater sensitivity for osseous lesions with cross-sectional imaging when compared to radiography and supported the guidelines of the International Myeloma Working Group which state that whole body CT may replace bone survey [8, 9]. Conventional radiography also lacks the functional information that can be provided by PET/CT, which is now recommended as the preferred method of monitoring therapy response given its ability to differentiate metabolically active from inactive disease [10]. PET/CT and MRI detect bone damage earlier than bone survey, and posttreatment metabolic activity on PET/CT is an important prognostic factor. Comparison of baseline versus posttreatment PET/CT metabolic activity is increasingly important in the setting of novel highly active agents, as it allows for identification of patients with minimal residual disease negativity.

## Figures and Tables

**Table 1:** Extraosseous myeloma distribution.

Site	Lesions	%
Upper airways, sinuses, and oral cavity	858	33.8
Central nervous system, head and neck	405	16.0
Soft tissue (not extremities)	357	14.1
Gastrointestinal	262	10.3
Thoracic	192	7.6
Reticuloendothelial system	164	6.5
Skin (not extremities)	154	6.1
Genitourinary	60	2.4
Extremities	44	1.7
Not otherwise specified	42	1.7

Total	2538	100.0

**Table 2:** Comprehensive table of extraosseous myeloma lesion distributions.

*Extremities*	*44*

Arm (skin)	1
Buttocks (SQ)	1
Fingers	2
Forearm	2
Gastrocnemius	1
Gluteus maximus	2
Knee	1
Knee (SQ)	1
Leg (skin)	1
Leg (soft tissue)	4
Leg (SQ)	1
Lower extremity	6
Median nerve	1
Shoulder girdle	2
Sural nerve	1
Thigh	7
Thigh (skin)	1
Thigh (SQ)	1
Toes	1
Triceps (SQ)	1
Upper extremity	6

*Gastrointestinal*	*262*

Colon	11
Duodenum	9
Esophagus	1
Gallbladder	1
“GI tract NOS”	45
“Intra-abdominal lesions”	8
Jejunum	2
Liver	67
Mesentery	6
Omentum	3
Pancreas	31
Pelvic mass	7
Peritoneum	31
Rectum	3
“Small bowel NOS”	8
Stomach	29

*GU*	*60*

Bladder	5
Kidney	26
Lower urinary tract	1
Ovary	6
Penis	1
Prostate	1
Scrotum	1
Testicle	13
Uterus	1
Vagina	3
Vulva	2

*CNS, head and neck*	*405*

Ears	1
“Eye or brain”	41
“Head and Neck NOS”	64
“Intracranial or Head/neck NOS”	6
Middle ear	2
Parapharyngeal space	3
Parotid gland	15
Preauricular region	1
Pterygoid fossa	1
Submandibular gland	3
“Submaxillary gland”	1
Supraclavicular fossa	4
Thyroid	23
Conjunctiva	3
Eyelid	5
Lacrimal gland	2
Orbit	19
CNS	196
Retrobulbar	15

*RES*	*164*

Lymph node	141
Spleen	23

*Skin*	*154*

Abdomen	1
Cheek	6
Cutaneous NOS	130
Nose	1
Scalp	10
Sternum	3
Within scars	3

*Soft tissue*	*357*

“Muscle NOS”	5
“Old catheter sites”	1
“Soft tissue NOS”	123
“Surrounding axial skeleton”	105
Abdomen	13
Adrenal	9
Axilla	2
Bone marrow biopsy site	1
Breast	28
Groin	1
Hips	1
Iliopsoas	5
Infratemporal fossa	1
Inguinal	2
Neck	9
Paravertebral	11
Periadrenal fat	1
Perirenal	5
Presacral	1
Retroperitoneum	23
Subcutaneous NOS	8
Trunk	2

*Thoracic*	*192*

Aorta	1
Chest/thoracic wall	45
Diaphragm	1
Heart	5
Lung	65
Mediastinum	18
Pericardium	6
Pleura + pleural effusion	51

*Other NOS*	*42*

*Upper airway*	*858*

Tongue	13
Cricoid	2
Epiglottis	3
Ethmoid sinus	16
False vocal cord	1
Gingiva/gums	24
Larynx	47
Lips	1
Maxillary sinus + maxilla + antrum	61
“Mouth/pharynx NOS”	115
Nares	5
Nasal cavity/fossa/mucosa	56
Nasal polyp	1
Nasal septum	6
Nasooropharynx	2
Nasopharynx	103
Nose	39
Oropharynx	25
“Palate/gum/maxilla NOS”	11
Paranasal sinus + sinus	37
Pharynx	19
“Respiratory NOS”	133
Rhinopharynx	4
Sinonasal/nasal sinus	41
Soft palate + palate + hard palate	4
Sphenoid sinus	15
Subglottic	5
Supraglottic	2
Thyroid cartilage	7
Tonsil	49
Trachea	3
Vocal cords	4
“Upper respiratory tract NOS”	4
